# Physical activity, black carbon exposure, and DNA methylation in the *FOXP3* promoter

**DOI:** 10.1186/s13148-017-0364-0

**Published:** 2017-06-13

**Authors:** Stephanie Lovinsky-Desir, Kyung Hwa Jung, Jacqueline R. Jezioro, David Z. Torrone, Mariangels de Planell-Saguer, Beizhan Yan, Frederica P. Perera, Andrew G. Rundle, Matthew S. Perzanowski, Steven N. Chillrud, Rachel L. Miller

**Affiliations:** 1 Division of Pediatric Pulmonology, Department of Pediatrics, College of Physicians and Surgeons, Columbia University, 3959 Broadway CHC-745, New York, NY 10032 USA; 2 Division of Pulmonary, Allergy and Critical Care of Medicine, Department of Medicine, College of Physicians and Surgeons, Columbia University, PH8E-101, 630 W. 168 St, New York, NY 10032 USA; 3 Department of Neurology, Columbia University Medical Center, 710 W. 168th St, New York, NY 10032 USA; 4 Lamont-Doherty Earth Observatory, Columbia University, 61 Rt, 9 W Palisades, New York, 10964 NY USA; 5 Department of Environmental Health Sciences and Columbia Center for Children’s Environmental Health, Mailman School of Public Health, Columbia University, 722 W. 168 St, New York, NY 10032 USA; 6 Department of Epidemiology, Mailman School of Public Health, Columbia University, 722 W. 168 St, New York, NY 10032 USA; 7 Division of Pediatric Allergy, Immunology, and Rheumatology, Department of Pediatrics, College of Physicians and Surgeons, Columbia University, PH8E-101, 630 W. 168 St, New York, NY 10032 USA

**Keywords:** Exercise, Air pollution, Buccal cells, Treg, Lung function, Spirometry, Biomarker

## Abstract

**Background:**

Physical activity is associated with improvement in lung function; however, pollution exposure during physical activity can lead to a transient reduction in lung function. This paradoxical relationship may be linked to altered T regulatory (Treg) cell activity, which increases with exercise and suppresses airway inflammation, but decreases in association with exposure to air pollution. To clarify these relationships, we investigated buccal cell DNA methylation of the forkhead box p3 (*FOXP3*) gene promoter, a proposed biomarker of Treg activity. We hypothesized that active urban children would have lower *FOXP3* promoter methylation, associated with better lung function compared to non-active children. We also hypothesized that this relationship would be attenuated by high exposure to the air pollutant black carbon (BC).

**Methods:**

We performed a cross-sectional study of 135 children ages 9–14 who live in New York City. Activity was measured across 6 days. BC exposure was assessed by personal monitors worn for two 24-h periods, followed by lung function assessment. Buccal swabs were collected for DNA methylation analysis of three regions (six CpG sites) in the *FOXP3* promoter.

**Results:**

In multivariable regression models, overall, there was no significant relationship between physical activity and *FOXP3* promoter methylation (*p* > 0.05). However, in stratified analyses, among children with higher BC exposure (≥1200 ng/m^3^), physical activity was associated with 2.37% lower methylation in promoter 2 (CpGs −77, −65, and −58) (*β*_estimate_ = −2.37%, *p* < 0.01) but not among those with lower BC exposure (*β*_estimate_ = 0.54%, *p* > 0.05). Differences across strata were statistically significant (*p*_interaction_ = 0.04). Among all children, after controlling for BC concentration, promoter 2 methylation was associated with reduced FEV_1_/FVC (*β*_estimate_ = −0.40%, *p* < 0.01) and reduced FEF_25–75%_ (*β*_estimate_ = −1.46%, *p* < 0.01).

**Conclusions:**

Physical activity in urban children appeared associated with lower *FOXP3* promoter methylation, a possible indicator of greater Treg function, under conditions of high BC exposure. Reduced *FOXP3* promoter methylation was associated with higher lung function. These findings suggest that physical activity may induce immunologic benefits, particularly for urban children with greater risk of impaired lung function due to exposure to higher air pollution. *FOXP3* promoter buccal cell methylation may function as a useful biomarker of that benefit.

**Electronic supplementary material:**

The online version of this article (doi:10.1186/s13148-017-0364-0) contains supplementary material, which is available to authorized users.

## Background

While regular physical activity is believed to reduce the frequency of asthma symptoms [1], exposure to air pollution is a known trigger of asthma [2, 3]. Some studies suggest that engaging in physical activity in polluted areas may reduce lung function [4, 5], increase airway inflammation [6], and increase the risk of developing asthma [7]. One possible explanation is that increased minute ventilation during exertion could lead to increased pollutant deposition in the lungs [8, 9]. Yet, the complex relationship between the benefits of regular physical activity and the risk associated with pollution exposure is not well understood.

Physical activity and exercise may improve overall health and lung function, particularly in individuals with asthma, by suppressing pro-allergic immune responses via the T regulatory (Treg) cell pathway [10, 11]. In support of this, Lowder et al. used an allergic asthma mouse model to demonstrate that moderate intensity activity over 4 weeks resulted in increased number and function of Tregs isolated from the lung and mediastinal lymph nodes [11]. Similarly, in a study of adults, increased Treg polarization of lymphocytes was observed with regular physical activity over a 12-week period while there was no change in a less active control group [12]. In contrast, reduced Treg cell number and function has been linked to environmental pollution exposure. For example, Nadeau and colleagues reported significant impairment in Treg function among children with asthma in the highly polluted region of Fresno, California, compared to children living in the less polluted Stamford, California region [13]. The same group also linked impaired Treg function to high exposure to the polycyclic aromatic hydrocarbon (PAH) class of air pollutants [14]. Furthermore, both animal [15, 16] and human studies [17] have demonstrated that Tregs suppress airway inflammation and airway hyper-responsiveness in allergic asthma. Thus, altered Treg cell activity appears to underlie how exposure to pollution impacts the relationship between physical activity and airway disease.

Activation of the forkhead box p3 (*FOXP3*) transcription factor controls the differentiation and function of Treg cells [18]; thus, *FOXP3* expression is an established marker of committed Treg cell populations and function [19]. Several studies have demonstrated that *FOXP3* is regulated through epigenetic mechanisms [20, 21]. For example, in vitro models have established that demethylation of the *FOXP3* promoter is critical to the stable expression of *FOXP3* and the suppressive function of Tregs [22]. Among ex vivo Treg cells, increased *FOXP3* promoter methylation has been inversely correlated with *FOXP3* expression [23]. In addition, increased *FOXP3* methylation has been linked directly to higher pollutant exposures including diesel exhaust particles [24], polycyclic aromatic hydrocarbons (PAHs) [13, 14], and secondhand smoke (SHS) [23]. Black carbon (BC), like PAHs, is a component of particulate matter (PM) and a major element of “soot” which is an incomplete combustion product emitted from diesel exhaust. Similar to SHS, BC exposure has been associated with respiratory symptoms [25], airway inflammation [26], and reduced lung function [27]. Thus, BC exposure also may contribute to the pollution-associated increase in *FOXP3* methylation and Treg impairment.

Our primary objective was to determine the interaction between physical activity and BC exposure on *FOXP3* promoter methylation in a cross-sectional sample of 9–14-year-old children that live in New York City (NYC). An additional objective was to determine the relationship between *FOXP3* promoter methylation and measures of lung function. We assessed *FOXP3* methylation in buccal cells as a surrogate for airway epithelium. *FOXP3* expression in buccal epithelium has been demonstrated by others [28], and *FOXP3* methylation in saliva has been linked previously to both exposure to pollution and asthma outcomes [24]. While physical activity may induce improved immune function through increased Treg activity, increased lung deposition of pollutants during physical activity could mitigate this protective effect. Therefore, we hypothesized that overall, physical activity would be associated with lower *FOXP3* promoter methylation, but that this association would lessen among those with high BC exposure. We also hypothesized that lower *FOXP3* promoter methylation would be associated with higher lung function.

## Methods

### Study population

Study participants (*n* = 163) were enrolled from the Columbia Center for Children’s Environmental Health (CCCEH) birth cohort that is comprised of children living in Northern Manhattan and the South Bronx of NYC, whose non-smoking, African American and Dominican mothers were recruited during pregnancy [29, 30]. Participants were recruited for this nested study based on age (target 9-14-year-olds) and current asthma. Asthma diagnosis was determined by a physician (allergist or pulmonologist) using standardized criteria during at least one cohort study visit between ages 5 and 12 years [31]. In addition, in order to be eligible for the nested study, children with asthma had to have report of asthma symptoms or asthma medication use in the 12 months prior to enrollment in the nested study. Total serum immunoglobulin E (IgE) was measured at age 9 or 11. Children with a total IgE level ≥80 IU/mL were considered seroatopic. Children with body mass index (BMI) percentiles ≥the 85th percentile for age and sex were classified as “overweight.” Secondhand smoke exposure (SHS) was determined by answering yes to the question, “In the last 2 weeks have you been exposed to tobacco smoke in the home.” Complete data on physical activity, BC exposure, DNA methylation, and lung function were available for *n* = 135 children that were included in this study. The longitudinal birth cohort study is conducted in accordance with the Columbia University Institutional Review Board guidelines, and written informed consents and assents were obtained.

### Physical activity assessment

All study participants wore an accelerometer (Actical, Philips Respironics, Bend, OR) continuously on the non-dominant wrist for six consecutive days (Additional file 1: Figure S1). To account for 24-h data that were partially collected on the first and last day, only the five intervening consecutive days with full 24-h of data were used for analysis [6, 32]. The accelerometer uses a sensor to detect and integrate amplitude and frequency of motion and stores the data as activity counts. Based on the activity counts, activity energy expenditure is determined with a cut point of <0.031 kcal/min/kg for light activity, >0.083 kcal/min/kg for vigorous activity, and moderate intensity activity in between. Summary statistics derived from the Actical software were used to quantify the amount of time spent in moderate and vigorous activity for each 24-h period. Physical activity was characterized based on the Center for Disease Control and Prevention (CDC) recommendation that children should participate in at least 60 min of moderate-to-vigorous activity (MVA) on a daily basis (http://www.cdc.gov/physicalactivity/everyone/guidelines/children.html). Children that met the physical activity recommendation, having at least 60 min of MVA daily, were considered “active” while children that did not meet the physical activity recommendation were considered “non-active” as previously published [6].

### Personal BC monitoring

Personal exposure to BC was measured over two 24-h periods at the beginning and end of the week-long physical activity monitoring period (Additional file 1: Figure S1). Children carried a MicroAeth (Model AE51, Magee Scientific, Berkley, CA) inside a vest pocket that contained an air inlet in the breathing zone (vest collar). Children were instructed to remove the vest during vigorous activity and to keep it nearby and uncovered. We previously have demonstrated excellent compliance with wearing the BC monitoring equipment [33]. BC was sampled from the air every 5 min and data were cleaned according to algorithms developed to account for false positive and negative measures that can result from physical vibration [34, 35]. Every 5 min, data were then averaged to yield a mean 24-h exposure assessment. Mean 24-h personal exposure to BC weakly correlated across the 2 days of measurement (Spearman *r* = 0.35, *p* < 0.01), reflecting some day to day variation in exposure. Therefore, personal BC exposure levels were averaged across the two 24-h monitoring periods and dichotomized at the median (1210 ng/m^3^) to represent high vs. low average exposure across the week. Fifty-three percent of the personal BC exposure measurements (*n* = 71) were sampled during the NYC cold weather heating season (i.e., from October to April).

### FOXP3 DNA methylation analysis

Buccal samples were collected using the CytoSoft Cytology brush [36, 37] (Fisher Scientific, Pittsburgh, PA, USA) from each child at two time points, 5 days apart corresponding to the 2 days of BC exposure assessment (Additional file 1: Figure S1). Samples contained approximately 94% squamous epithelial cells, determined by slide smear hematoxylin and eosin stain, as previously published [38]. Bisulfite conversion was performed on 200 ng of genomic buccal cell DNA using Zymo Research’s EZ DNA Methylation-Lightning Kit (Irvine, CA, USA) as previously published [37, 38].

Polymerase chain reaction (PCR) primers and pyrosequencing primers (Additional file 2: Table S1) were designed using PyroMark Assay Design 2.0 software (Qiagen, Valencia, CA, USA) to target six CpG sites in the promoter region of the *FOXP*3 gene. The promoter region was defined as the gene region directly upstream of the transcription start site (TSS) (Additional file 1: Figure S2). The individual CpG sites were selected based on previous literature that demonstrated methylation in this region was associated with ambient air pollution exposure [13]. We chose to focus on CpG sites in the gene promoter which is the principal activator of *FOXP3* expression [22] as opposed to CpG sites in the Treg-cell-specific demethylated region (TSDR) which play a role in stabilizing *FOXP3* expression [39] and are only activated in Treg cells [40]. PCR reactions were performed with Qiagen Hot Star Taq DNA polymerase reagents (Qiagen Sciences, Germantown, MD, USA) with the following concentrations for each ingredient in the PCR mixtures: 1× PCR buffer, 1.5 μM MgCl_2_, 200 μM dNTP, 0.2 μM forward primer, and 0.2 μM reverse primer. PCR was performed under the following conditions: 95 °C, 5 min; 45 cycles of 95 °C, 30 s; 57 °C, 1 min; 72 °C, 1 min; 72 °C, 10 min; and 4 °C hold. The PCR product was sequenced using PyroMark Q96 Pyrosequencer. EpiTect high and low methylated control DNA (Qiagen Sciences, Germantown, MD, USA) were included with every pyrosequencing experiment.

Three distinct promoter regions were defined by the proximity to neighboring CpG sites: promoter 1 included CpGs −138 and −126; promoter 2 included CpGs −77, −65, and −58; and promoter 3 included CpG −15 (Additional file 1: Figure S2). Percent methylation was moderately correlated across each promoter region (*r* = 0.22−0.45, *p* < 0.01). Therefore, percent methylation was averaged across the two CpG sites in promoter 1 and the three CpG sites in promoter 2. Percent methylation measured across the two separate days was moderately correlated for each of the promoter regions (*r* = 0.47−0.68, *p* < 0.01) (Additional file 2: Table S2). Therefore, in order to account for short-term variability in methylation across the 1 week of physical activity monitoring [36], methylation was averaged across the 2 days. For subjects in which there was only 1 day of methylation data available because of a laboratory technical failure (*n* = 8 for promoter 1 and *n* = 2 for promoter 2), the one available methylation value was used in place of an average.

### FOXP3 mRNA expression analysis

A separate buccal swab was collected from each child and stored in RNA*later* solution (Qiagen Sciences, Germantown, Maryland, United States). RNA was extracted using the Trizol method (Invitrogen, Life Technologies Europe BV, Monza, Italy) as previously published [37]. RNA concentration and purity was measured using a NanoDrop spectrophotometer (Thermo Scientific, Wilmington, DE, USA). Complementary (cDNA) synthesis of up to 200 μg of RNA was transcribed with a SuperScript First-Strand Synthesis System for RT-PCR (Invitrogen, Life Technologies Europe BV, Monza, Italy) according to the manufacturer’s instructions. Quantitative real-time PCR was performed using a 25-μl reaction volume containing 2 μl cDNA template, 12.5 μl SYBR Green Mix (Applied Biosystems, Foster City, CA, USA), 9.5 μl H20, and 0.2 μM of forward and reverse primer (Additional file 2: Table S1). Amplifications were performed in duplicate with an initial incubation at 95 °C for 30 s, followed by 40 cycles of 95 °C for 10 s and 55 °C for 30 s, using a CFX Connect Real-Time PCR Detection System (Bio-Rad, Hercules, CA, USA).

*FOXP3* mRNA expression levels were determined using the 2^−∆Ct^ method with cystatin A (CSTA) as a reference gene (highly and stably expressed in non-malignant epithelial tissue [41]) and normalized to the lowest measured value. Across the 2 days of sampling, relative mRNA expression was moderately correlated (*r* = 0.31, *p* < 0.01, Additional file 2: Table S2); therefore, values were averaged across the 2 days. For the subjects for which there was insufficient template RNA and cDNA to perform real-time PCR experiments for one of the measurement days (*n* = 6), we used a single value in place of a 2-day average yielding a total sample size of 132 for all RNA analysis.

### Pulmonary function assessment

Spirometry was used to assess pulmonary function during in-home visits on days 1 and 6, immediately following BC assessment (Additional file 1: Figure S1). Studies were performed using a portable spirometer (Koko, nSpire Health, Longmont, CO, USA), in accordance with ATS and ERS guidelines [31]. Four spirometry outcome measures were used for this analysis, forced vital capacity (FVC), forced expiratory volume in 1 s (FEV_1_), the ratio of FEV_1_/FVC, and the forced expiratory flow at 25–75% (FEF_25–75%_). Spirometry results were interpreted independently by two pulmonologists to ensure acceptability criteria were met based on ATS and ERS guidelines [42].

### Statistical analysis

Chi-square and *t* tests were used to explore differences in demographic characteristics between the active and non-active children. Spearman correlations were used to assess methylation correlations across promoter regions. Kruskal-Wallis tests were used to determine the difference in *FOXP3* promoter methylation between active vs. non-active children and between high vs. low BC concentrations.

Step-wise multivariable linear regression models were fit to examine the association between physical activity and *FOXP3* promoter methylation with active (coded 1) vs. non-active (coded 0) as the main predictor of interest. Models were further stratified by high vs. low BC concentrations (dichotomized at the median). To assess for interaction, we introduced a cross product term of activity x BC concentration to our non-stratified models. Because *FOXP3* promoter methylation was not normally distributed, we also performed a sensitivity analysis using ordinal logistic regression where methylation was categorized by quartiles. To examine the association of *FOXP3* promoter methylation on lung function, we again performed step-wise linear regression models with percent methylation as the predictors of interest and FVC, FEV_1_, FEV_1_/FVC, and FEF_25–75%_ percent predicted as the outcomes.

To explore potential associations between activity, pollution, and *FOXP3* expression, in the secondary analysis, we substituted relative mRNA expression for methylation in our above mentioned models. We also performed a secondary analysis to examine the relationship between physical activity and lung function. As an exploratory analysis, we further stratified our activity-lung function model by the upper tertile of BC concentration (≥1790 ng/m^3^) to isolate the children with the most extreme BC exposure concentrations. For these exploratory analyses, we chose to stratify by the upper tertile of BC based on our previous findings that physical activity was associated with decrease airway inflammation but not among children with the highest exposure to BC (upper tertile) [6]. Lastly, given that the *FOXP3* gene is located on the X-chromosome and may be susceptible to X-chromosome inactivation (XCI) resulting in sex differences in methylation patterns [43, 44], we performed ancillary analyses stratified by sex. All final models were adjusted for the following covariates: age, sex (except in models stratified by sex), race/ethnicity, height (lung function models only), body mass index (BMI) Z-score [45], asthma, atopy (total IgE ≥80 IU/mL), personal BC concentration (except in the models stratified by BC), SHS exposure, and heating season.

All statistical analyses were performed using SAS 9.4.

## Results

### Subject characteristics

Demographic characteristics for the 135 children included in this analysis are provided in Table 1. The children in the active group were slightly younger than the children in the non-active group (*p* < 0.01). There were fewer children in the non-active group with SHS exposure compared to children in the active group (*p* = 0.04). Average time spent (±SD) in MVA across the 5-day observation period among active children was 210 min/day (±84) and among non-active children was 101 min/day (±58).

**Table 1:** Participant characteristics for the *n* = 135 children included in this study

	Non-active (*n* = 58)	Active (*n* = 77)	*p* value^a^
Age in years, median (range)	12.7 (10.5–14.0)	12.2 (9.2–14.0)	*<0.01*
Males, *n* (%)	32 (55%)	35 (45%)	0.26
Race/ethnicity, *n* (%) Hispanic African American	42 (72%) 16 (28%)	46 (60%) 31 (40%)	0.13
Asthma^b^, *n* (%)	30 (52%)	45 (58%)	0.44
FVC % predicted, mean (SD)	85% (12.1)	86% (10.5)	0.92
FEV_1_ % predicted, mean (SD)	84% (13.0)	85% (11.6)	0.72
FEV_1_/FVC % predicted, mean (SD)	87% (7.5)	87% (5.3)	0.52
FEF_25–75%_ % predicted, mean (SD)	83% (24.2)	86% (21.7)	0.36
Total IgE ≥80 IU/mL	31 (53%)	38 (49%)	0.64
BMI ≥85th percentile, *n* (%)	31 (53%)	39 (51%)	0.75
Secondhand smoke^c^, *n* (%)	3 (5%)	14 (18%)	*0.04*
Heating season^d^, *n* (%)	25 (43%)	44 (57%)	0.11
Black carbon, GM (SD)	1070 (1.69)	1190 (1.78)	0.25
High BC^e^, *n* (%)	26 (45%)	37 (48%)	0.71

*FVC* forced vital capacity, *FVE*_*1*_ forced expiratory volume in 1 s, *FEF*_*25–75%*_ forced expiratory flow at 25–75%, *IgE* Immunoglobulin E, *BMI* body mass index, *BC* black carbon

^a^Chi-square test for categorical variables and *t* test for continuous variables (age and black carbon)

^b^Determined by a physician at age 5–12 years using a priori standardized criteria

^c^Determined by responding yes to the question “In the last 2 weeks have you been exposed to tobacco smoke in the home.” missing *n* = 1 from the active group

^d^Observation period took place during NYC’s cold weather heating season (October to April)

^e^Dichotomized at the median, 1210 ng/m^3^

Italicised values in the final column represent a *p*-value < 0.05

### Independent associations of physical activity and BC exposure on FOXP3 methylation

**Fig. 1:**
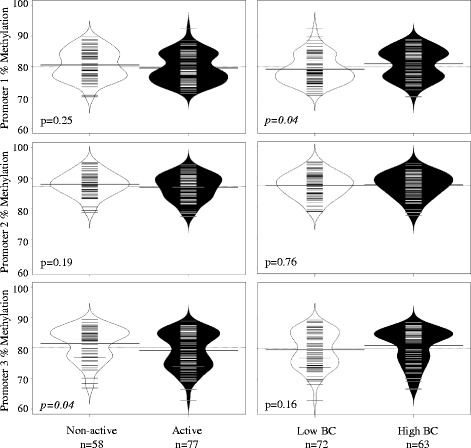
Distribution of *FOXP3* promoter methylation stratified by active vs. non-active children and by low vs. high black carbon (Kruskal-Wallis test). The *short lines* indicate individual observations, while the shaded areas indicated the distribution of the data for each respective group. The *dotted lines* indicate the mean percent methylation for all participants, and the *thicker solid lines* represent mean percent methylation for each respective group

Buccal cell methylation in the *FOXP3* promoter regions ranged from 62.9 to 95.1% (promoter 1: mean ± SD, 80.0 ± 4.8; promoter 2: 87.4 ± 4.0; promoter 3: 80.2 ± 5.9). We observed moderate correlations in *FOXP3* methylation across the three promoter regions (*r*_spearman_ 0.56–0.58, *p* < 0.01) (Additional file 1: Figure S3). In bivariate analysis, active children had lower promoter 3 methylation (79.2 ± 6.1) compared to non-active children (81.4 ± 5.4, *p* = 0.04) (Fig. 1). Also, children with high personal BC exposure had higher promoter 1 methylation compared to children with low BC (80.9 ± 4.8 vs. 79.1 ± 4.7, *p* = 0.04) (Fig. 1).

In multivariable linear regression models, we did not observe an association between physical activity and *FOXP3* promoter methylation (*p* = 0.31, 0.33, and 0.35 for promoters 1, 2, and 3, respectively) (Table 2).

**Table 2:** Active children with high exposure to BC have lower *FOXP3* promoter methylation compared to non-active children with high BC

	Difference in percent methylation in active vs. non-active children *β*_estimate_ (95% confidence interval)			
	Overall sample (*n* = 135)	High BC^a^ (*n* = 63)	Low BC^a^ (*n* = 72)	*p*_interaction_
Promoter 1	−0.59 (−1.72, 0.54)	−1.32 (−2.74, 0.11)	0.30 (−1.36, 1.97)	NA
Promoter 2	−0.60 (−1.79, 0.60)	*−2.37 (−4.04, −0.70)*	0.54 (−1.12, 2.20)	*0.04*
Promoter 3	−0.68 (−2.10, 0.74)	*−2.57 (−4.62, −0.51)*	−0.41 (−2.16, 1.34)	0.26

*β*_estimate_ represents the effect size or the difference in percent methylation when comparing active children to non-active children (reference). Models adjusted for age, sex, race/ethnicity, BMI Z-score, asthma, atopy, secondhand smoke exposure, BC (only in non-stratified model), and heating season. Italicized values represent *p* value ≤0.01 and *p* < 0.05 for interaction term. *P*_interaction_ represents the *p* value for the interaction term between physical activity and BC

^a^Dichotomized at the median, 1210 ng/m^3^

### Combined association of physical activity and BC exposure on FOXP3 methylation

**Fig. 2:**
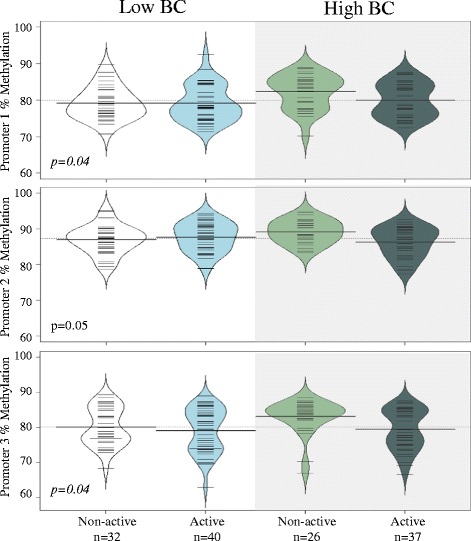
Non-active children with high BC on average have the highest *FOXP3* promoter methylation (Kruskal-Wallis test). The *short lines* indicate individual observations, while the *shaded areas* indicated the distribution of the data for each respective group. The *dotted lines* indicate the mean percent methylation for all participants, and the *thicker solid lines* represent mean percent methylation for each respective group

We first compared *FOXP3* promoter methylation across four groups of children (non-active/low BC, active/low BC, non-active/high BC, and active/high BC) (Fig. 2). Non-active children with high exposure to BC had the highest methylation in all *FOXP3* promoter sites (*p* < 0.05, Fig. 2). We next performed multivariable linear regression models to examine the association between physical activity and *FOXP3* methylation stratified by high vs. low BC concentrations. Among children with high personal BC measures (*n* = 63), active children on average had 2.4% lower promoter 2 methylation (*β*_estimate_ [95% CI], −2.37 [−4.04, −0.70], *p* < 0.01) and 2.6% lower promoter 3 methylation (−2.57 [−4.62, −0.51], *p* = 0.01) compared to non-active children (Table 2). There was no significant association between physical activity and *FOXP3* methylation among children with low personal BC concentration (*p* > 0.05) (Table 2). We observed a significant interaction in the association between activity and promoter 2 methylation by BC concentration (*p*_interaction_ = 0.04), but not with promoter 3 methylation (*p*_interaction_ = 0.26) (Table 2).

In sensitivity analysis using ordinal logistic regression, we again observed a trend towards an inverse association between activity and methylation among children with high BC (*p* = 0.09, 0.06, and 0.07 for promoters 1, 2, and 3, respectively) (Additional file 2: Table S3). There was no association between activity and methylation among children with low BC (*p* > 0.05).

### Association between FOXP3 methylation and lung function

**Fig. 3:**
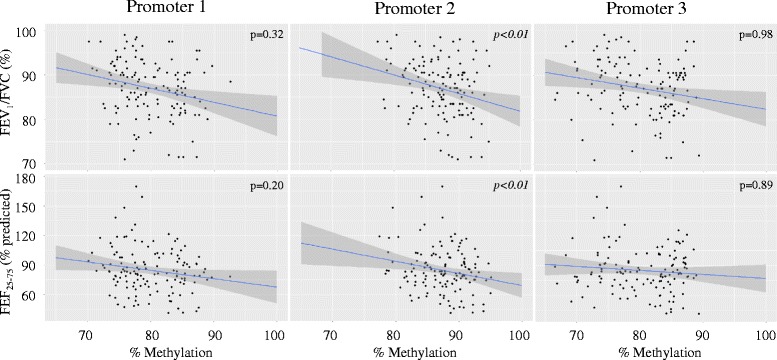
Lower *FOXP3* promoter methylation is associated with higher FEV_1_/FVC and FEF_25_−_75%_. The *blue line* represents the mean effect estimate, and the *shaded gray* area represents the 95% confidence interval (*n* = 135)

We next examined the association between *FOXP3* promoter methylation and lung function outcomes. There was an inverse association between *FOXP3* promoter 2 methylation and both the FEV_1_/FVC and FEF_25–75%_ (Fig. 3). On average, for every 10% increase in *FOXP3* promoter 2 methylation, there was a 4% decrease in FEV_1_/FVC (*β*_estimate_ [95% CI], −0.40 [−0.67, −0.13], *p* < 0.01). Similarly, for every 10% increase in *FOXP3* promoter 2 methylation, there was a 15% decrease in FEF_25–75%_ (−1.46 [−2.52,−0.40], *p* < 0.01) (Additional file 2: Table S4). Also, in our crude models, there were inverse associations between promoter 1 methylation and both FEV_1_/FVC (−0.18 [−0.30, −0.05], *p* < 0.01) and FEF_25–75%_ (−0.04 [−0.07, −0.00], *p* = 0.03); however, these findings were not statistically significant after controlling for confounders. There was no significant association between promoter 3 methylation and lung function nor between methylation and FVC or FEV_1_. Also, the relationship between promoter 2 methylation and lung function did not significantly vary by high vs. low BC exposure (Additional file 2: Table S5).

### Secondary analyses

First, we performed substitution analysis with *FOXP3* relative expression. However, we did not observe a correlation between *FOXP3* methylation of any of the three promoter sites with relative mRNA expression (promoter 1: *r* = −0.03, *p* = 0.74; promoter 2: *r* = −0.10, *p* = 0.24; promoter 3: *r* = 0.05, *p* = 0.57). Nonetheless, in the secondary analyses, we substituted *FOXP3*-relative mRNA expression in our models to explore the relationship with combined activity and BC concentrations as well as lung function. There was no significant association between physical activity and mRNA expression in the high (*β*_estimate_ [95% CI], 0.02 [−0.00, 0.05], *p* = 0.11) or low (−0.00 [−0.01, 0.00], *p* = 0.12) personal BC groups. There was no significant association between relative mRNA expression and lung function (data not shown).

Second, we explored the overall relationship between physical activity and lung function using multivariable linear regression models. We observed a significant positive association between physical activity and FEF_25–75%_. On average, active children had 8% greater FEF_25–75%_ compared to non-active children (8.07 [0.40, 15.74], *p* = 0.04) (Table 3). To explore relationships among the children with the most extreme pollutant measures and highest risk based on previous findings [6], we further stratified our model by the upper tertile of BC concentration. Interestingly, among children with the highest BC exposure, active children had lower FEV_1_ and FVC compared to non-active children. Conversely, among children with lower BC exposure, active children had higher FEV_1_ and FEF_25–75%_ compared to non-active children (Table 3). However, there was no significant interaction between activity and BC exposure on any of the lung function outcomes (Table 3). Lastly, in ancillary analyses stratified by sex, we observed that *FOXP3* promoter methylation was lower in females compared to males (*p* < 0.01, Additional file 1: Figures S4 and S5), and the relationships between activity, *FOXP3* promoter methylation, and lung function were stronger among females compared to those among males (Additional file 2: Table S6, Additional file 2: Table S7).

**Table 3:** Among children with the upper tertile of exposure to BC, physical activity is associated with reduced lung function, whereas children with less BC exposure experience opposite effects

	Difference in methylation in active vs. non-active children *β*_estimate_ (95% confidence interval)			
	Overall sample (*n* = 135)	Upper tertile BC^a^ (*n* = 33)	Lower 2/3 BC^a^ (*n* = 102)	*p*_interaction_
FEV_1_	2.72 (−1.14, 6.59)	*−7.87 (−15.33, −0.42)*	*4.60 (0.21, 8.99)*	0.09
FVC	1.25 (−2.25, 4.75)	*−6.91 (−13.70, −0.13)*	2.70 (−1.30, 6.70)	0.31
FEV_1_/FVC	1.36 (−0.62, 3.33)	−1.09 (−5.03, 2.5)	1.90 (−0.49, 4.28)	NA
FEF_25−75%_	*8.07 (0.40, 15.74)*	−6.59 (−23.0, 9.80)	*9.60 (0.60, 18.60)*	0.27

*β*_estimate_ represents the effect size or the difference in percent methylation when comparing active children to non-active children (reference). Models adjusted for age, sex, race/ethnicity, BMI Z-score, asthma, atopy, secondhand smoke exposure, heating season, and BC concentration (only for overall sample model). Italicized values represent *p* value ≤0.05. *P*_interaction_ represents the *p* value for the interaction term between physical activity and BC

^a^Dichotomized at the upper tertile, 1790 ng/m^3^

## Discussion

In our cohort of 9–14-year-old children of African American and Dominican descent living in NYC, we identified a link between higher combined personal BC exposure and physical activity and lower *FOXP3* promoter methylation. In addition, *FOXP3* promoter methylation was inversely associated with FEV_1_/FVC and FEF_25–75%_, the two indicators of airway obstructive physiology [46, 47]. These findings suggest that higher exposure to air pollution may be a condition by which urban children are more likely to gain immunological benefits of regular exercise, and that *FOXP3* promoter methylation may function as a biomarker of that benefit.

Our a priori hypothesis was that active children would have lower *FOXP3* buccal cell methylation compared to non-active children. Our findings were consistent with this hypothesis only within the promoter 3 region, which is the CpG site most proximal to the transcription start site. An additional hypothesis was that high BC exposure, which has been associated with adverse respiratory outcomes [25–27], would attenuate the association between physical activity and methylation. Contrary to our hypothesis, the association between physical activity and *FOXP3* methylation was only notable among children with high BC exposure. This may instead suggest that, although physical activity may increase lung deposition of ambient pollutants and thereby increase airway inflammation, a high degree of regular physical activity may induce sufficient protective immune balance to mitigate this effect. *FOXP3* promoter methylation levels among active children with high BC were comparable to both groups of children with low BC (active and non-active) (Fig. 2). Thus, we speculate that children with higher risk of Treg impairment due to high exposure to pollutants are the ones able to achieve greater immunologic benefit from regular physical activity. Fisher and colleges recently investigated the interaction between physical activity and nitrogen dioxide (NO_2_) pollutant exposure on respiratory-related hospitalization in a cohort of Danish adults [48]. The authors reported no significant interaction between physical activity and pollutant exposure on hospitalizations for asthma suggesting that the benefits of activity outweighed the risk of pollutant exposure. Through our ability to assess recent activity and acute air pollution with high granularity, our findings further suggest a significant short-term positive interaction between pollutant exposure and activity on immune function, as indicated by *FOXP3* promoter methylation.

Divergent from previous reports in humans [23], we were unable to detect a correlation between buccal *FOXP3* promoter methylation and relative mRNA expression. One possible explanation is that the above mentioned relationship previously has been identified in isolated Treg cells while we sampled buccal cells. Also, currently, there is no standardized method for quantifying methylation across CpG sites within a gene region, thus limiting the ability to compare percent methylation levels across various studies [13, 23]. To our knowledge, this is one of the first studies to investigate *FOXP3* methylation in buccal epithelium; however, our absolute percent methylation levels are comparable to those measured in gingival epithelial cells [49]. Methylation patterns can differ by cell type [37, 50], which is likely reflective of lower mRNA expression of immunomodulatory genes in buccal epithelium vs. higher expression among lymphocytes. We previously reported higher methylation in buccal cells compared to CD4+ lymphocytes within the interferon gamma (*IFNγ*) immunomodulatory gene promoter. Additionally, a significant correlation in *IFNγ* methylation across the cell types was notable for one of the CpG sites [37], suggesting a link between buccal cell and lymphocyte methylation in that particular gene. Buccal cells are easily accessible, apt for repeat testing with changing environmental exposures and less invasive than sampling the blood or lower airway epithelial cells. Thus, they may represent a suitable source of a biomarker for pediatric cohort research.

Notably, our most significant findings were observed in the promoter 2 region that encompasses two of three CpG sites (−77 and −65) that are conserved in humans, mice, and rats [22]. Others also have reported that individual CpG sites within a gene region may be critical drivers of downstream effects [51, 52]. Our methylation signal was small, and our observed differences in *FOXP3* promoter methylation between active and non-active children was about 2.5% [51, 52]. Breton and colleagues also described small differences in buccal cell methylation of several genes between children that were exposed vs. those not exposed to secondhand smoke [53]. Nonetheless, the observation that buccal cells were not completely methylated may suggest that we are capturing a small signal that could reflect larger differences among immune-specific cells. In addition, our observations between methylation and lung function suggest that small changes in buccal epithelial methylation alone could be clinically meaningful. This is consistent with a recent review article that appraised the clinical significance of small-magnitude effect sizes in pediatric environmental epigenetic research, similar to those reported in this current study [54].

Further, the *FOXP3* gene is located on the X-chromosome and thus, may be susceptible to X-chromosome inactivation (XCI) in females. This too may be driven and maintained by DNA methylation [44]. However, sex differences in methylation attributed to XCI can vary by location within a gene [55]. In our cohort, we observed lower *FOXP3* promoter methylation in females compared to males (Additional file 1: Figures S4 and S5), consistent with the findings in a different cohort of children. In that cohort, differences were more striking and in the opposite direction within the *FOXP3* enhancer region [43]. In this current study, the association between physical activity and *FOXP3* methylation among children with high BC was most notable in females compared to males (Additional file 1: Figures S6 and S7, Additional file 2: Table S6). Likewise, the relationship between *FOXP3* promoter methylation and lung function was stronger in the females (Additional file 2: Table S7). Overall, these findings underscore the importance of considering sex effects in methylation studies, particularly within genes that are susceptible to XCI.

Breton and colleagues also identified a significant inverse relationship between buccal cell DNA methylation of another asthma gene, arginase (ARG)2, and airway inflammation measured by fractional exhaled nitric oxide (FeNO) [56]. Similarly, Baccarelli and colleagues identified an association between nasal epithelial cell methylation of inducible nitric oxide synthase (*iNOS*) and interleukin (*IL*)*6* with FeNO, but not with lung function (FEV_1_) [57]. While we too did not appreciate a significant association in methylation of our candidate genes, *FOXP3* and FEV_1_; we did, however, observe associations with FEV_1_/FVC and FEF_25–75%_. Both FEV_1_/FVC [46] and FEF_25–75%_ [47] have been demonstrated as better predictors of airway obstruction [47] and asthma severity [46] compared to FEV_1_. Furthermore, we observed that active children had higher FEF_25–75%_ compared to non-active children which supports our previous finding of a protective effect of physical activity on airway inflammation [6].

While we believe that our findings are fairly robust, several limitations of our study need to be acknowledged. First, our cross-sectional study design limits our ability to infer causality because of concerns regarding temporality. However, it is unlikely that one’s DNA methylation could cause a person to be more or less active or have greater or lesser exposure to air pollution. Also, in the current analysis DNA methylation was averaged across two measurements 5 days apart. This approach was taken to account for variation in methylation that can occur across short time intervals [36]. Also, while we compared *FOXP3* promoter methylation to *FOXP3* expression among buccal cells, we did not correlate buccal cell *FOXP3* methylation with Treg function directly. We acknowledge that our *FOXP3* buccal cell methylation is not equivalent for measuring altered Treg function related to physical activity, lung function, and exposure to pollutants in immune tissues. Rather, our findings suggest that *FOXP3* promoter methylation should be further explored as a biomarker of activity-mediated immune regulation. Generalizability of our findings is limited given our selected population of African American and Dominican children that live in an urban environment. However, one rationale for this selective cohort is that minority children in urban populations that we sampled here are at the greatest risk for asthma-associated morbidity [58, 59]. Similarly, the exploratory observations between physical activity and lung function that differed by high and low BC concentrations may be even more robust in a larger sample of children. However, the parent study was not designed or adequately powered to assess these relationships.

## Conclusions

We have demonstrated that combined physical activity and personal BC exposure may influence *FOXP3* promoter DNA methylation and that *FOXP3* promoter methylation is related to lung function. Our study has identified a unique subgroup of children with high BC exposure that may benefit the most from regular physical activity. While the mechanisms are unknown, these findings suggest that exercise may be associated with a protective immune response in the setting of high pollution exposure. *FOXP3* promoter methylation may be a useful biomarker of this protection. Overall, our findings are supportive of both the use of buccal cells for DNA methylation studies as well as a possible role of DNA methylation in respiratory outcomes.

## Additional files

Additional file 1: Figure S1. Sampling scheme for accelerometer, black carbon (BC), buccal swabs for DNA and RNA analysis and spirometry. **Figure S2.** Schematic representation of the *FOXP3* gene and the six CpG sites in the promoter region that were investigated. *TSS* transcription start site, *TSDR* Treg-specific demethylated region, *CNS* conserved non-coding sequence. **Figure S3.** Correlations of *FOXP3* methylation across promoter regions and with mRNA relative expression. **Figure S4.** Distribution of *FOXP3* promoter methylation in females vs. males stratified by physical activity (active vs. non-active). Females have lower *FOXP3* promoter methylation compared to males. **Figure S5.** Distribution of *FOXP3* promoter methylation in females vs. males stratified by BC concentration (low vs. high). Females have lower *FOXP3* promoter methylation compared to males. **Figure S6.** Distribution of *FOXP3* promoter methylation stratified by combined activity and BC concentration in females (*n* = 67). **Figure S7.** Distribution of *FOXP3* promoter methylation stratified by combined activity and BC concentration in males (*n* = 68).

Additional file 2: Table S1. Primers for PCR and pyrosequencing experiments. **Table S2.** Correlations of day 1 vs. day 6 *FOXP3* methylation and mRNA expression. **Table S3.** Among children with high BC exposure, there is a trend towards active children (coded 1) having a greater odds of lower methylation compared to non-active children (coded 0). **Table S4.** Higher *FOXP3* promoter 2 methylation is associated with overall lower lung function (*n* = 135). **Table S5.** The relationship between *FOXP3* promoter 2 methylation and lung function does not significantly vary by high vs. low BC exposure. **Table S6.** Among children with high BC, the association between physical activity and *FOXP3* promoter methylation is greater in females. **Table S7.** The relationship between *FOXP3* promoter methylation and lung function is greater among females compared to that among males.

## Abbreviations

ARG: Arginase
BC: Black carbon
BMI: Body mass index
CCCEH: Columbia Center for Children’s Environmental Health
CSTA: Cystatin A
FEF_25–75%_: Forced expiratory flow at 25–75%
FeNO: Fractional exhaled nitric oxide
FEV_1_: Forced expiratory value in 1 s
FOXP3: Forkhead box p3
FVC: Forced vital capacity
IFNγ: Interferon gamma
IgE: Immunoglobulin E
IL6: Interleukin 6
iNOS: Inducible nitric oxide synthase
MVA: Moderate-to-vigorous activity
NO_2_: Nitrogen dioxide
NYC: New York City
PAH: Polycyclic aromatic hydrocarbon
PCR: Polymerase chain reaction
PM: Particulate matter
SHS: Second hand smoke
Treg: Regulatory T cell
TSDR: Treg-cell-specific demethylated region
XCI: X-chromosome inactivation
