# Supplementary material for: Physical activity, black carbon exposure, and DNA methylation in the FOXP3 promoter
Source: Clin Epigenetics. 2017 Jun 13;9:65. doi: 10.1186/s13148-017-0364-0 (PMC5470266; doi:10.1186/s13148-017-0364-0)
Supplement: Supplementary file 1 — Sampling scheme for accelerometer, black carbon (BC), buccal swabs for DNA and RNA analysis and spirometry. Figure S2. Schematic representation of the FOXP3 gene and the six CpG sites in the promoter region that were investigated. TSS transcription start site, TSDR Treg-specific demethylated region, CNS conserved non-coding sequence. Figure S3. Correlations of FOXP3 methylation across promoter regions and with mRNA relative expression. Figure S4. Distribution of FOXP3 promoter methylation in females vs. males stratified by physical activity (active vs. non-active). Females have lower FOXP3 promoter methylation compared to males. Figure S5. Distribution of FOXP3 promoter methylation in females vs. males stratified by BC concentration (low vs. high). Females have lower FOXP3 promoter methylation compared to males. Figure S6. Distribution of FOXP3 promoter methylation stratified by combined activity and BC concentration in females (n = 67). Figure S7. Distribution of FOXP3 promoter methylation stratified by combined activity and BC concentration in males (n = 68). [file 13148_2017_364_MOESM1_ESM.pptx]

## Slide 1
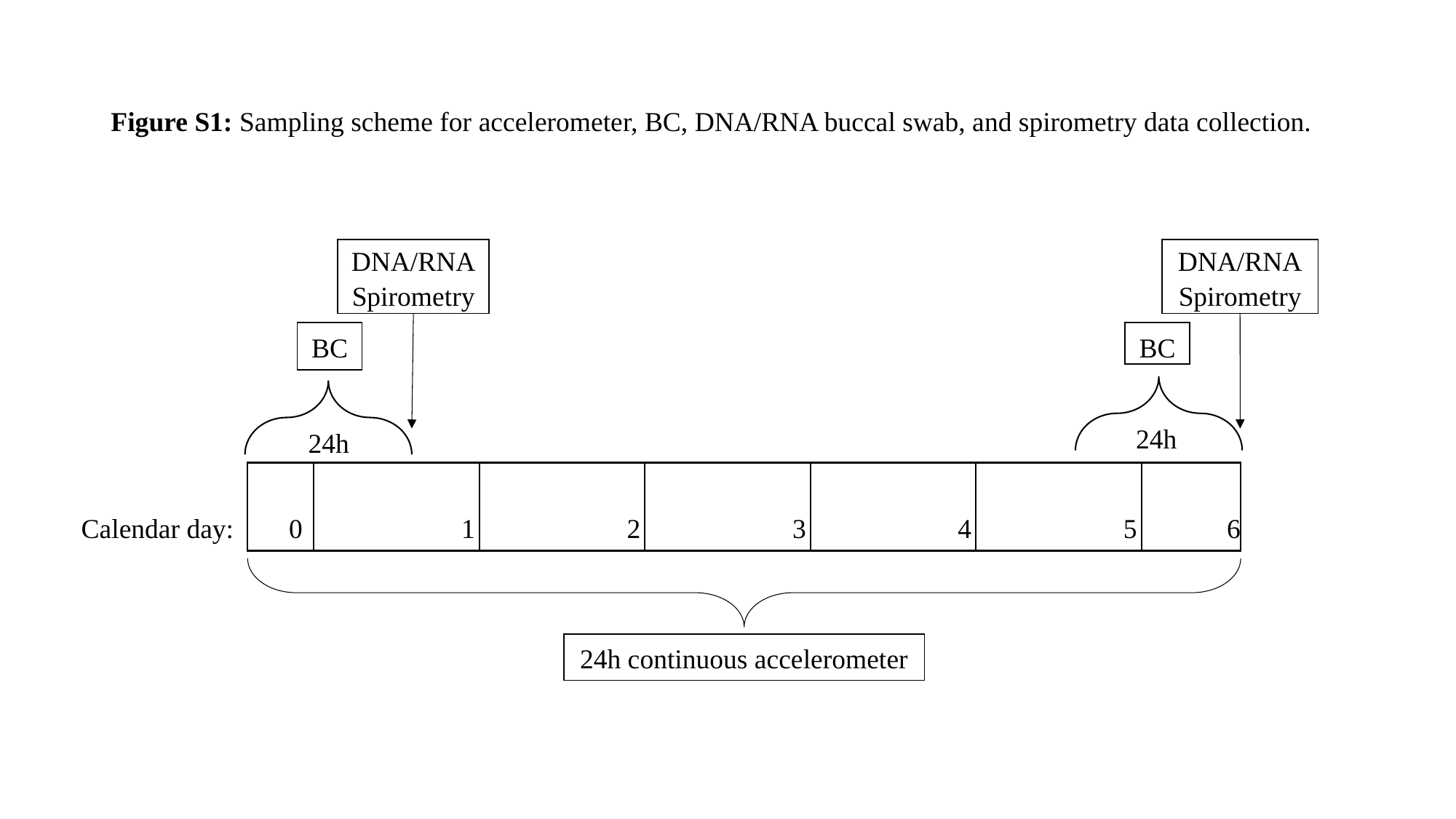

# Figure S1: Sampling scheme for accelerometer, BC, DNA/RNA buccal swab, and spirometry data collection.
DNA/RNA
Spirometry
DNA/RNA
Spirometry
BC
BC
24h
24h
Calendar day: 0 1 2 3 4 5 6
24h continuous accelerometer

## Slide 2
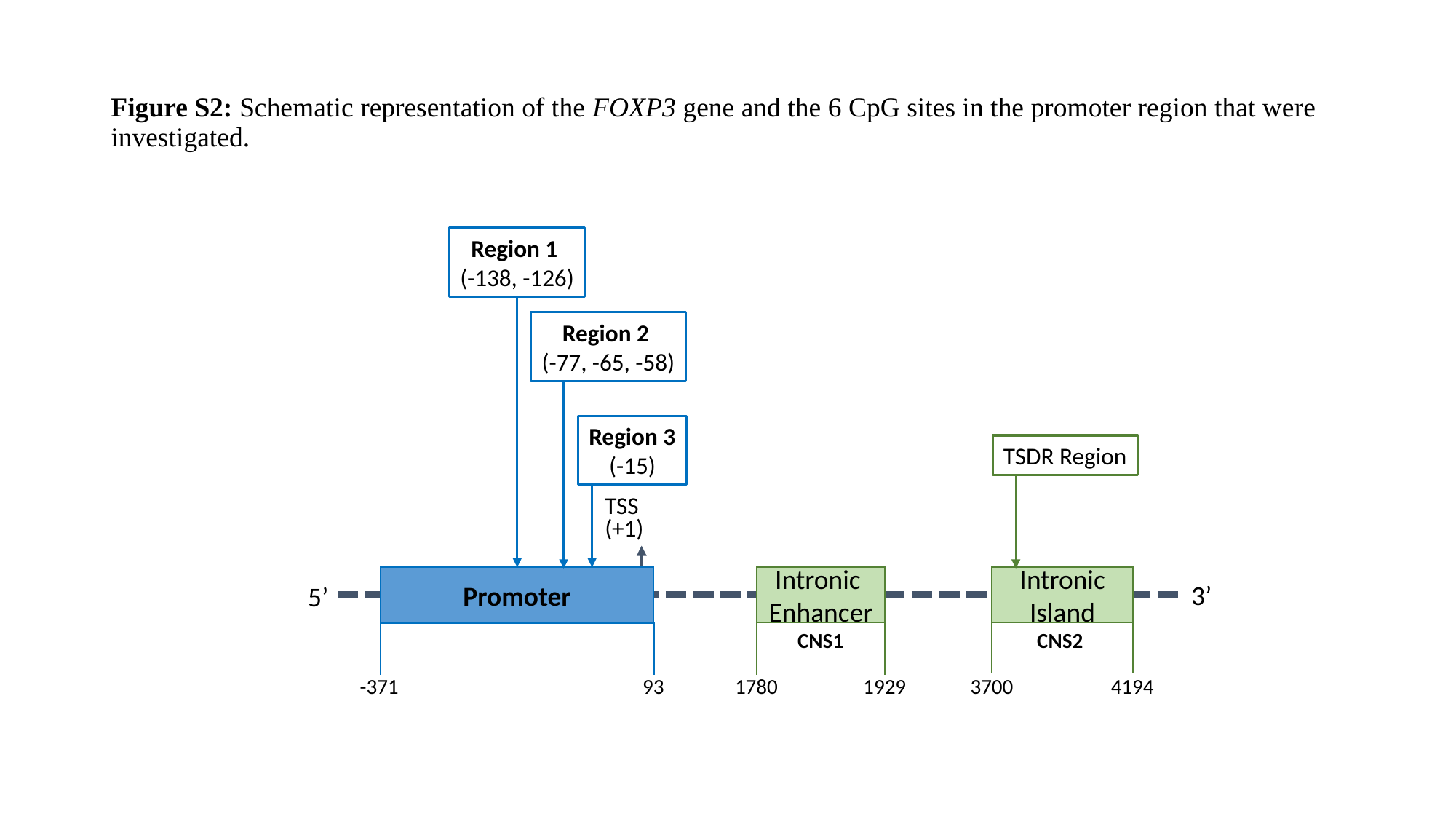

# Figure S2: Schematic representation of the FOXP3 gene and the 6 CpG sites in the promoter region that were investigated.
Region 1
(-138, -126)
Region 2
(-77, -65, -58)
Region 3
(-15)
TSS
(+1)
Promoter
-371
93
TSDR Region
Intronic Enhancer
Intronic Island
3’
5’
CNS1
CNS2
1780
1929
3700
4194

## Slide 3
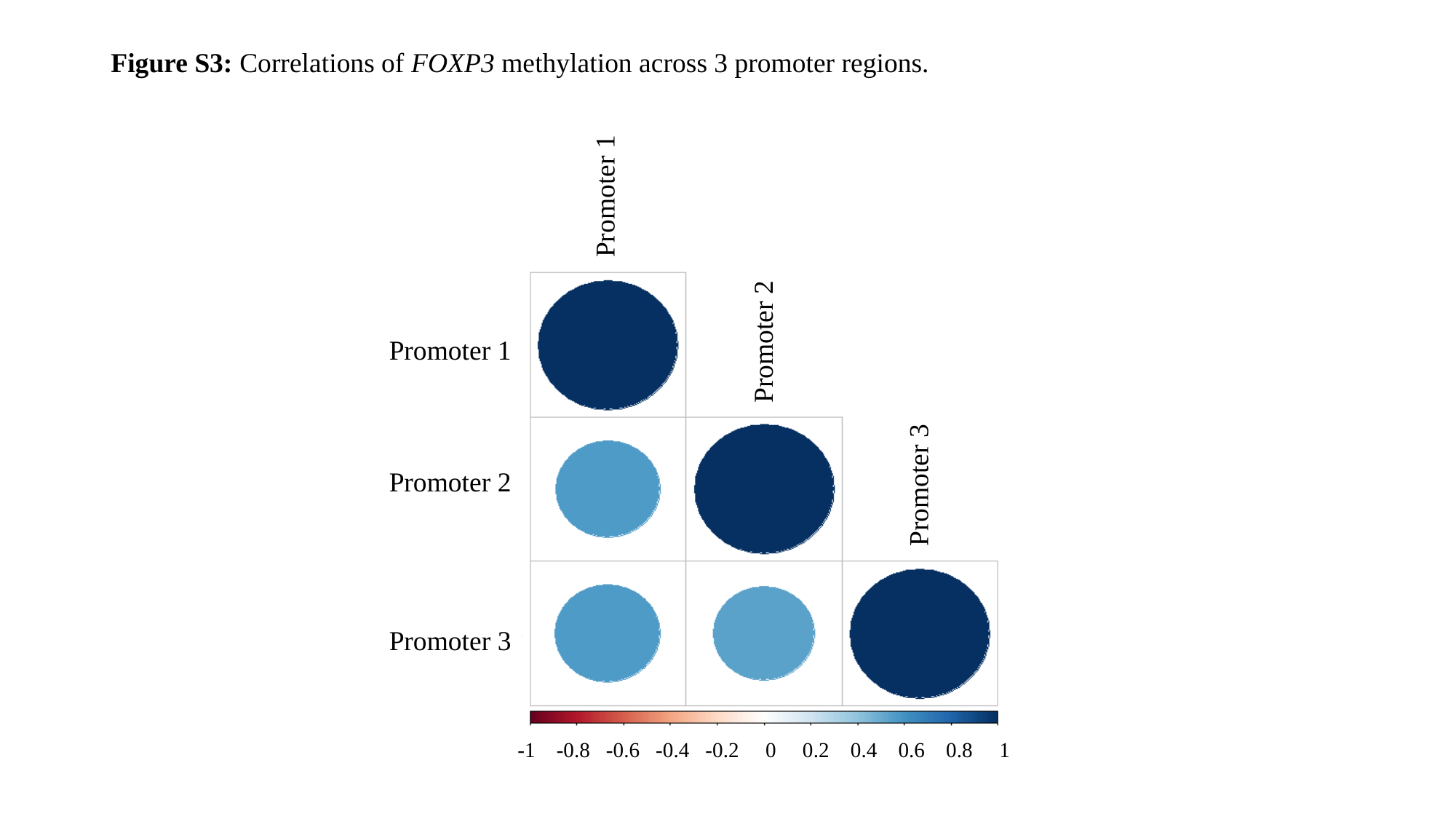

# Figure S3: Correlations of FOXP3 methylation across 3 promoter regions.
Promoter 1
Promoter 2
Promoter 1
Promoter 3
Promoter 2
Promoter 3
-1 -0.8 -0.6 -0.4 -0.2 0 0.2 0.4 0.6 0.8 1

## Slide 4
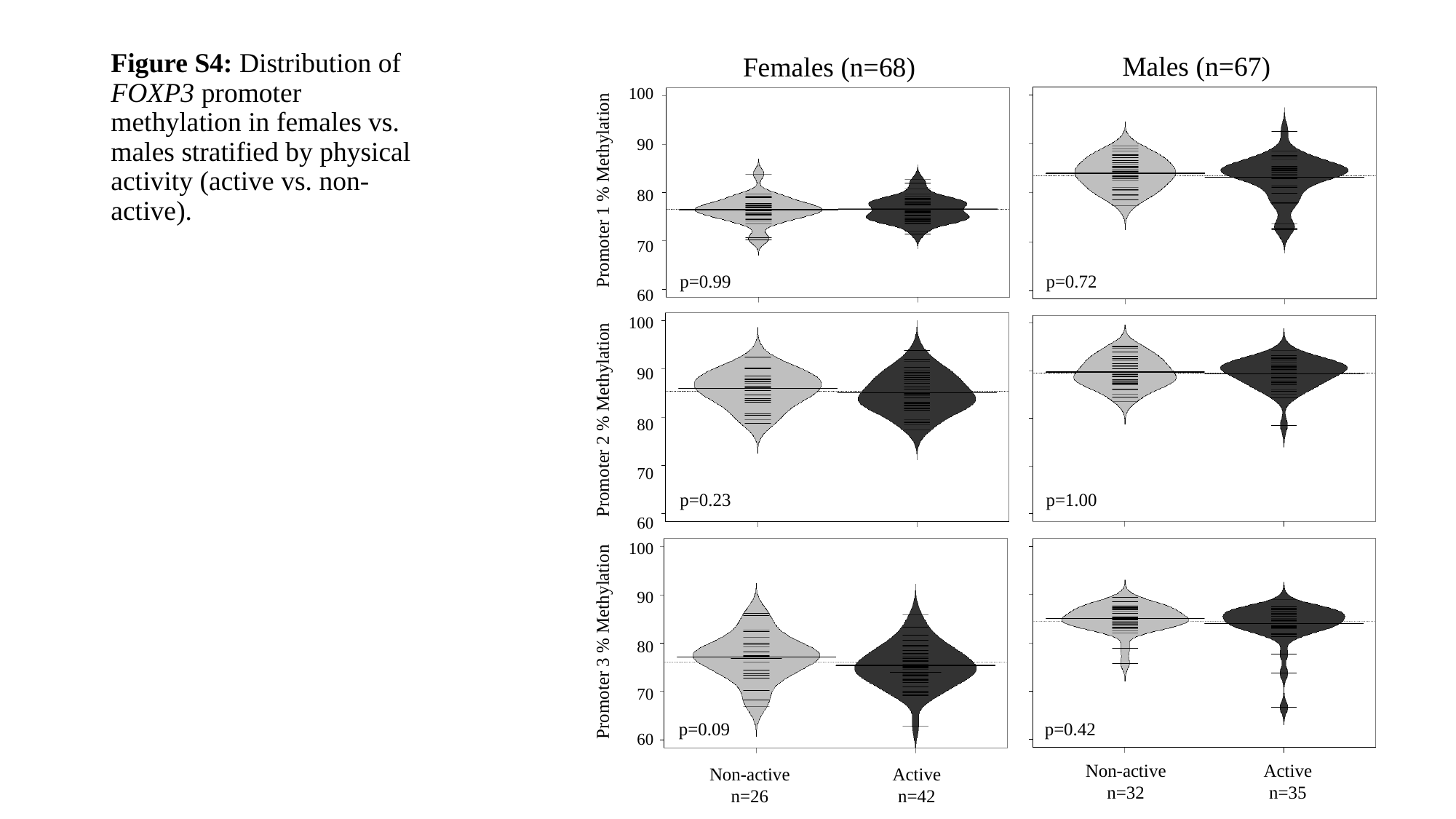

# Figure S4: Distribution of FOXP3 promoter methylation in females vs. males stratified by physical activity (active vs. non-active).
Males (n=67)
100
90
80
70
60
Promoter 1 % Methylation
p=0.99
100
90
80
70
60
Promoter 2 % Methylation
p=0.23
100
90
80
70
60
Promoter 3 % Methylation
p=0.09
Non-active
n=26
Active
n=42
Females (n=68)
p=0.72
p=1.00
p=0.42
Non-active
n=32
Active
n=35

## Slide 5
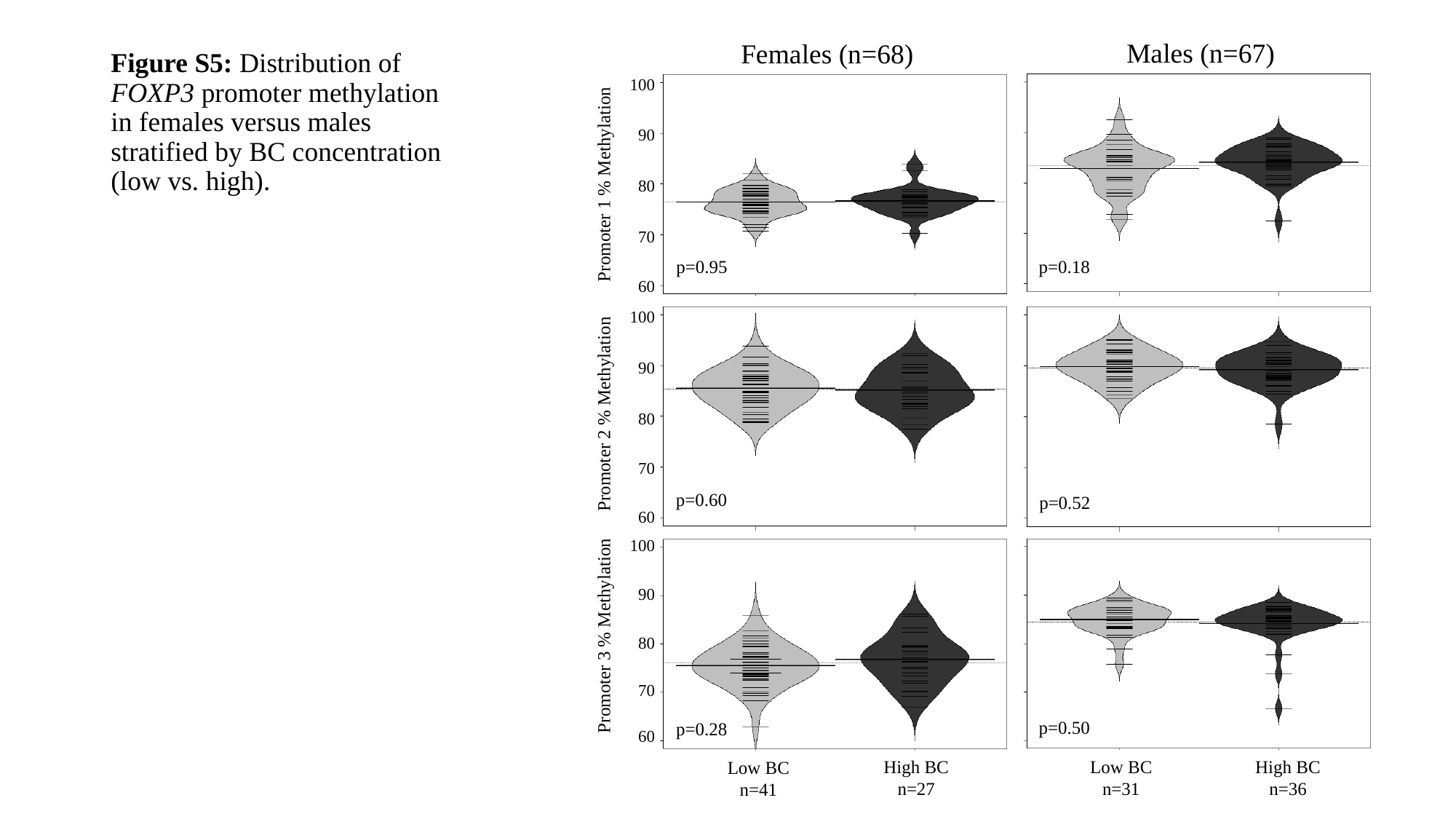

Males (n=67)
Females (n=68)
100
90
80
70
60
Promoter 1 % Methylation
p=0.95
100
90
80
70
60
Promoter 2 % Methylation
p=0.60
100
90
80
70
60
Promoter 3 % Methylation
p=0.28
High BC
n=27
Low BC
n=41
p=0.18
p=0.52
p=0.50
Low BC
n=31
High BC
n=36
# Figure S5: Distribution of FOXP3 promoter methylation in females versus males stratified by BC concentration (low vs. high).

## Slide 6
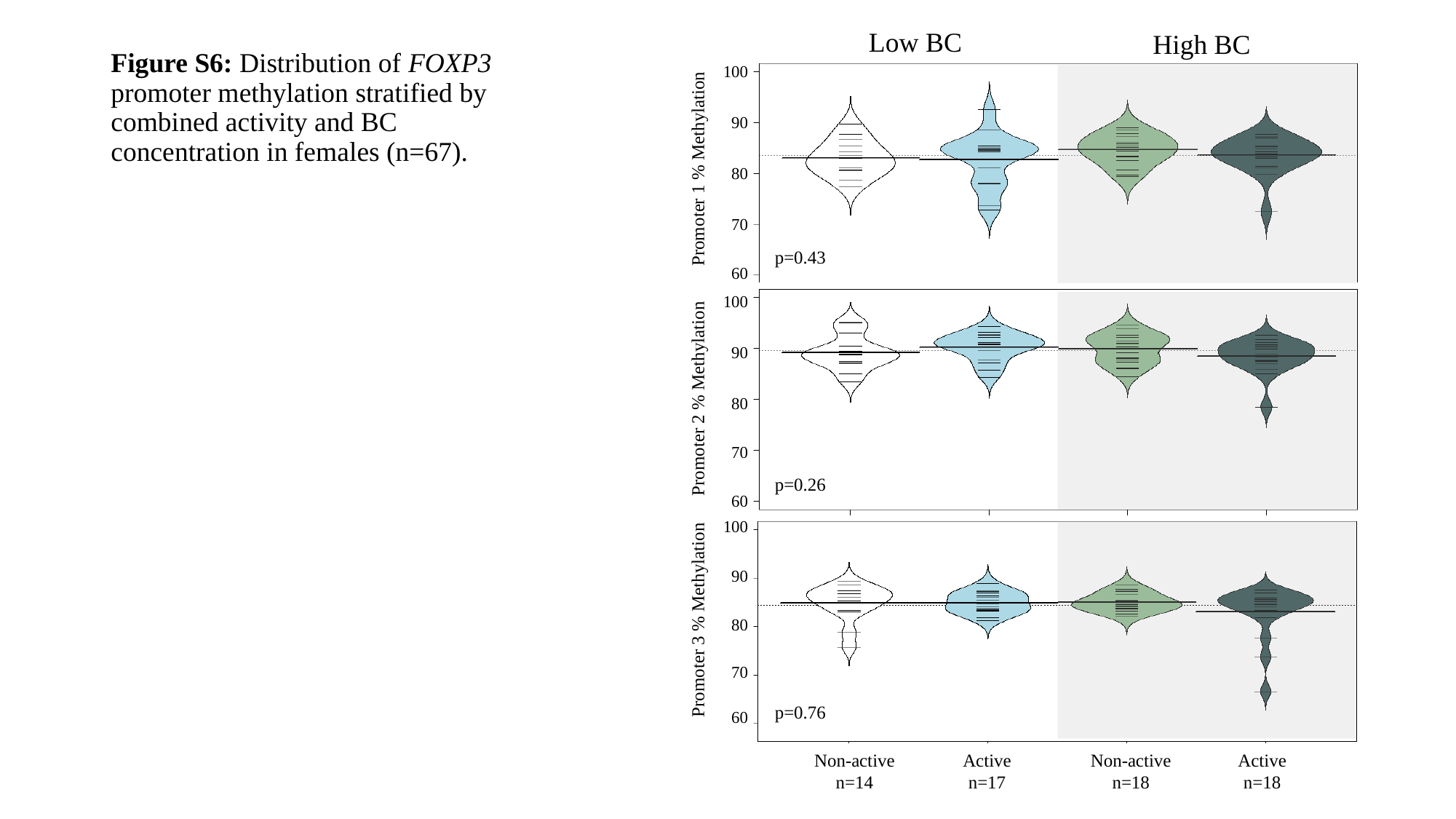

Low BC
High BC
100
90
80
70
60
Promoter 1 % Methylation
p=0.43
100
90
80
70
60
Promoter 2 % Methylation
p=0.26
100
90
80
70
60
Promoter 3 % Methylation
p=0.76
Active
n=17
Active
n=18
Non-active
n=14
Non-active
n=18
# Figure S6: Distribution of FOXP3 promoter methylation stratified by combined activity and BC concentration in females (n=67).

## Slide 7
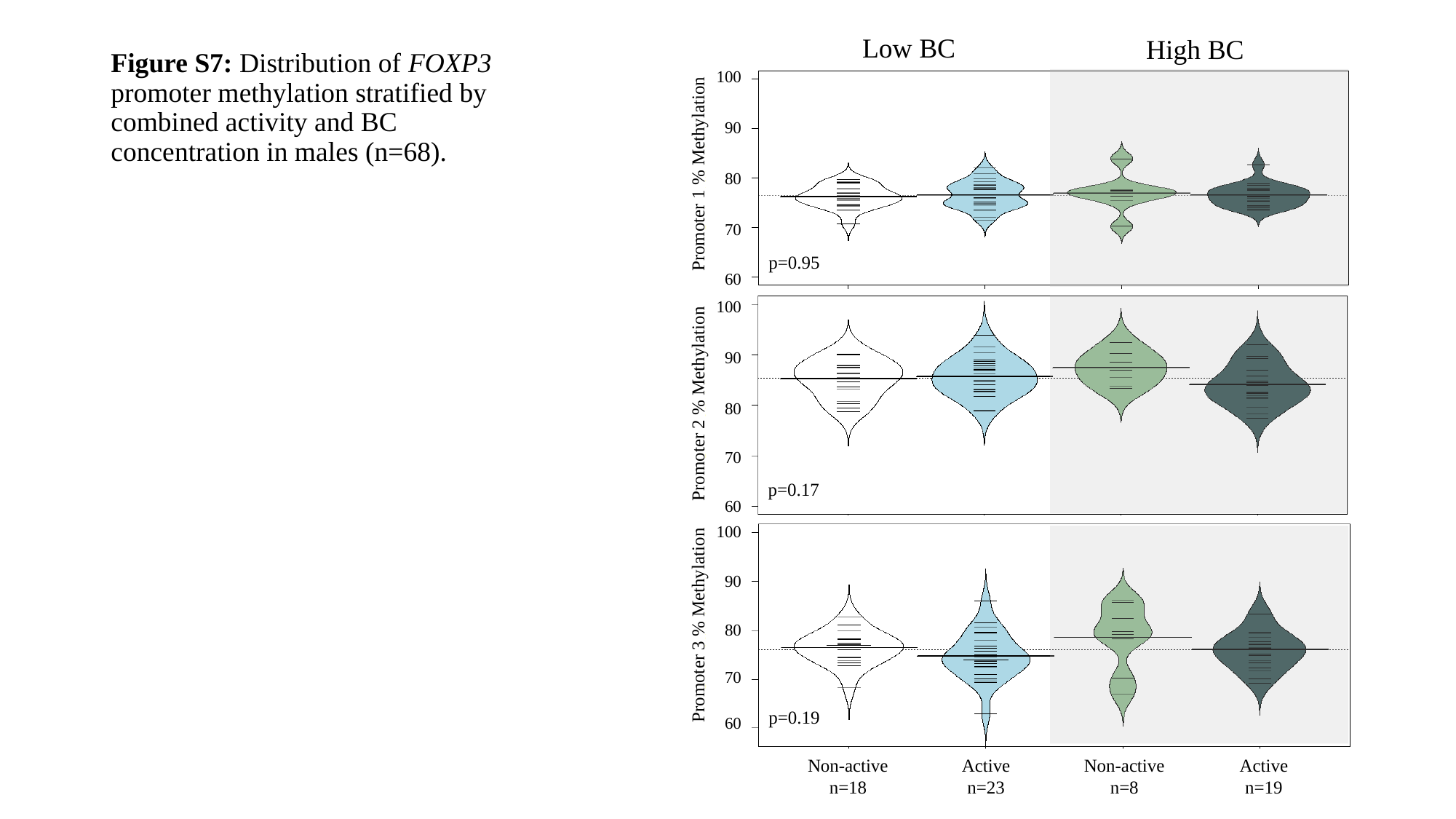

Low BC
High BC
100
90
80
70
60
Promoter 1 % Methylation
p=0.95
100
90
80
70
60
Promoter 2 % Methylation
p=0.17
100
90
80
70
60
Promoter 3 % Methylation
p=0.19
Active
n=19
Non-active
n=18
Active
n=23
Non-active
n=8
# Figure S7: Distribution of FOXP3 promoter methylation stratified by combined activity and BC concentration in males (n=68).
