# Supplementary material for: Physical activity, black carbon exposure, and DNA methylation in the FOXP3 promoter
Source: Clin Epigenetics. 2017 Jun 13;9:65. doi: 10.1186/s13148-017-0364-0 (PMC5470266; doi:10.1186/s13148-017-0364-0)
Supplement: Supplementary file 2 — Primers for PCR and pyrosequencing experiments. Table S2. Correlations of day 1 vs. day 6 FOXP3 methylation and mRNA expression. Table S3. Among children with high BC exposure, there is a trend towards active children (coded 1) having a greater odds of lower methylation compared to non-active children (coded 0). Table S4. Higher FOXP3 promoter 2 methylation is associated with overall lower lung function (n = 135). Table S5. The relationship between FOXP3 promoter 2 methylation and lung function does not significantly vary by high vs. low BC exposure. Table S6. Among children with high BC, the association between physical activity and FOXP3 promoter methylation is greater in females. Table S7. The relationship between FOXP3 promoter methylation and lung function is greater among females compared to that among males. [file 13148_2017_364_MOESM2_ESM.docx]

**Table S1:** Primers for PCR and pyrosequencing experiments

| Gene/region | Assay | Forward | Reverse | Sequencing |
| --- | --- | --- | --- | --- |
| *FOXP3* promoter 1:  CpGs -138, -126 | PCR and Pyrosequencing | 5’-[Biotin]-TTTTTGTGGTGAGGGGAAGAAATTA-3’ | 5’-  AACCCCAAACCTCTCTCTTCTAATAATCCA | 5’-CCAAATTTTTTTCCATAAATATAT-3’ |
| *FOXP3* promoter 2: CpGs -77, -65, -58 |  | 5’-AAATTTGGATTATTAGAAGAGAGAGG-3’ | 5’-[Biotin]-AACTAACAAAAAAAAATCAACCTAACTTAT-3’ | 5’-AGAAGAGAGAGGTTTG-3’ |
| *FOXP3* promoter 3: CpG -15 |  | 5’-AAATTTGGATTATTAGAAGAGAGAGG-3’ | 5’-[Biotin]-AACTAACAAAAAAAAATCAACCTAACTTAT-3’ | 5’-TTTTTAGGTATAAAAGTAAAGTTGT-3’ |
| *FOXP3* (target) | RT-qPCR | 5’- CGGACCATCTTCTGGATGAG-3’ | 5’-TTGTCGGATGATGCCACAG-3’ |  |
| *CSTA* (internal control) |  | 5’- AAACCCGCCACTCCAGAAATC-3’ | 5’- CACCTGCTCGTACCTTAATGTAG-3’ |  |

**Table S2:** Correlations of day 1 versus day 6 *FOXP3* methylation and mRNA expression.

|  | n | r_spearman_ | p value |
| --- | --- | --- | --- |
| Promoter 1 | 127 | 0.51 | <0.01 |
| Promoter 2 | 133 | 0.47 | <0.01 |
| Promoter 3 | 135 | 0.68 | <0.01 |
| mRNA Expression | 129 | 0.31 | <0.01 |

**Table S3:** Among children with high BC exposure there is a trend towards active children (coded 1) having a greater odds of lower methylation compared to non-active children (coded 0).

|  | Difference in methylation in active vs. non-active children | | | | | |
| --- | --- | --- | --- | --- | --- | --- |
|  | Overall sample  (n=135) | | High BC^ǂ^  (n=63) | | Low BC^ǂ^  (n=72) | |
|  | OR (95% CI) | p value | OR (95% CI) | p value | OR (95% CI) | p value |
| Promoter 1 | 1.38  (0.68, 2.82) | 0.37 | 2.74  (0.85, 8.84) | 0.09 | 0.81  (0.31, 2.17) | 0.68 |
| Promoter 2 | 1.02  (0.52, 2.03) | 0.95 | 2.76  (0.95, 7.99) | 0.06 | 0.56  (0.21, 1.48) | 0.24 |
| Promoter 3 | 1.30  (0.64, 2.64) | 0.48 | 2.79  (0.94, 8.34) | 0.07 | 1.51  (0.54, 4.22) | 0.43 |

Model adjusted for age, sex, race/ethnicity, BMI Z-score, asthma, atopy, second hand smoke exposure, BC (only in non-stratified model) and heating season.

ǂDichotomized at the median, 1,210 ng/m^3^.

**Table S4:** Higher *FOXP3* promoter 2 methylation is associated with overall lower lung function (n=135).

|  | Difference in methylation in active vs non-active children  β_estimate_ (95% Confidence Interval) | | | |
| --- | --- | --- | --- | --- |
|  | FVC | FEV_1_ | FEV_1_/FVC | FEF_25-75%_ |
| Promoter 1 | 0.03  (-0.49, 0.55) | -0.19  (-0.76, 0.39) | -0.15  (-0.44, 0.14) | -0.75  (-1.90, 0.39) |
| Promoter 2 | 0.23  (-0.26, 0.72) | -0.22  (-0.76, 0.33) | **-0.40**  **(-0.67, -0.13)** | **-1.46**  **(-2.52, -0.40)** |
| Promoter 3 | 0.21  (-0.20, 0.63) | 0.19  (-0.27, 0.65) | -0.00  (-0.24, 0.23) | 0.07  (-0.86, 0.99) |

Models adjusted for age, sex, race/ethnicity, height, BMI Z-score, asthma, atopy, BC concentration, second hand smoke exposure, and heating season.

**Bold** values represents p-value <0.01.

**Table S5:** The relationship between *FOXP3* promoter 2 methylation and lung function does not significantly vary by high vs. low BC exposure.

|  | β_estimate_ (95% Confidence Interval) | | |
| --- | --- | --- | --- |
|  | High BC^ǂ^  (n=63) | Low BC^ǂ^  (n=72) | p interaction |
| FEV_1_ | -0.35  (-0.34, 1.04) | 0.00  (-0.65, 0.66) | 0.74 |
| FVC | -0.05  (-0.74, 0.65) | -0.55  (-1.31, 0.21) | 0.75 |
| FEV_1_/FVC | -0.34  (-0.74, 0.06) | **-0.52**  **(-0.86, -0.19)** | 0.20 |
| FEF_25-75%_ | **-1.70**  **(-3.07, -0.33)** | **-1.60**  **(-3.09, -0.10)** | 0.24 |

Physical activity is a dichotomous predictor, therefore β_estimate_ represents the difference in the methylation outcome when comparing active children to non-active children (reference). Models adjusted for age, sex, race/ethnicity, BMI Z-score, height, asthma, atopy, second hand smoke exposure, BC and heating season.

ǂDichotomized at the median, 1,210 ng/m^3^.

**Bold** values represent p-value ≤0.05.

P-interaction represents the p-value for the interaction term between promoter 2 methylation and BC.

**Table S6:** Among children with high BC the association between physical activity and *FOXP3* promoter methylation is greater in females.

|  | Difference in methylation in active vs non-active children  β_estimate_ (95% Confidence Interval) | | |
| --- | --- | --- | --- |
|  | Overall sample with high BC  (n=63) | Females with high BC  (n=27) | Males with high BC  (n=36) |
| Promoter 1 | -1.32  (-2.74, 0.11) | **-2.11**  **(-4.13, -0.10)** | -1.69  (-3.71, 0.33) |
| Promoter 2 | **-2.37**  **(-4.04, -0.70)** | **-4.92**  **(-8.31, -1.53)** | -1.57  (-3.24, 0.10) |
| Promoter 3 | **-2.57**  **(-4.62, -0.51)** | **-3.72**  **(-7.18, -0.27)** | **-2.80**  **(-5.19, -0.41)** |

Models adjusted for age, sex, race/ethnicity, height, BMI Z-score, asthma, atopy, BC concentration, second hand smoke exposure, and heating season.

**Bold** values represents p-value <0.01.

**Table S7:** The relationship between *FOXP3* promoter methylation and lung function is greater among females compared to males.

|  | Difference in methylation in active vs non-active children  β_estimate_ (95% Confidence Interval) | | |
| --- | --- | --- | --- |
|  | Overall sample  (n=135) | Females  (n=68) | Males  (n=67) |
| FEV_1_/FVC | **-0.40**  **(-0.67, -0.13)** | **-0.44**  **(-0.76, -0.13)** | -0.29  (-0.77, 0.20) |
| FEF_25-75%_ | **-1.46**  **(-2.52, -0.40)** | **-1.51**  **(-3.00, -0.02)** | -1.21  (-2.77, 0.36) |

Physical activity is a dichotomous predictor, therefore β_estimate_ represents the difference in the methylation outcome when comparing active children to non-active children (reference). Models adjusted for age, sex, race/ethnicity, BMI Z-score, height, asthma, atopy, second hand smoke exposure, BC and heating season.

**Bold** values represent p-value ≤0.05.
